# The interplay between ovule number, pollination and resources as determinants of seed set in a modular plant

**DOI:** 10.7717/peerj.5384

**Published:** 2018-07-31

**Authors:** Marina M. Strelin, Marcelo A. Aizen

**Affiliations:** Laboratorio Ecotono, INIBIOMA (CONICET – Universidad Nacional del Comahue), San Carlos de Bariloche, Río Negro, Argentina

**Keywords:** Limits to plant reproduction, Ovule number, Physiological modularity, Pollination, Raspberry, Resources, Seed set

## Abstract

**Background:**

A classical dichotomous perspective proposes that either pollination or plant resources limit seed production. However, ovule number could also be limiting when pollination results in complete ovule fertilization and there are more plant resources available than needed to develop seeds. Moreover, this dichotomous view assumes that all flowers of a plant have equal access to a shared pool of resources, although these are frequently compartmentalized within plant modules, for example, inflorescences. How ovule number, pollination and resources affect seed production in physiologically-compartmentalized rather than physiologically-integrated plants has yet to be explored. We used raspberry (*Rubus idaeus*) to address this question.

**Methods:**

We first assessed if ovule number affected the fraction of ovules that develop into seed (i.e., seed set) and whether this effect related to the extent of physiological integration among flowers within plants. This was achieved by statistically testing predictions on the sign and level of plant organization (i.e., among flowers within inflorescences, among inflorescences within ramets, and among ramets) of the relation between ovule number and seed set given different degrees of physiological integration. We then explored whether the relation between ovule number and seed set was affected by plant age (used here as a surrogate of resource availability) and pollination intensity (open-pollination vs. exclusion).

**Results:**

Within inflorescences, flowers with more ovules set a larger fraction of seeds. On the other hand, seed set at the inflorescence level was negatively related to the average number of ovules per flower. Seed set increased with ovule number and open-pollination, and decreased with ramet age. However, ovule number explained more variation in seed set than ramet age and pollination treatment. Ramet age affected the strength of the relation of seed set to ovule number, which was stronger in old than young ramets. Pollination did not alter the strength of this relation to any significant extent.

**Discussion:**

Results reveal the importance of ovule number as an overriding factor affecting seed set. Within inflorescences, resources appear to be differentially allocated to developing fruits from flowers with many ovules. This is consistent with the fact that in the raspberry a large proportion of the carbon invested in fruit development is fixed by the inflorescence subtending leaf. Differential resource allocation to flowers with many ovules is not affected by pollinator exclusion, being stronger in resource-exhausted ramets. This suggests that the effects of pollen limitation and resource allocation are compartmentalized at the inflorescence level. Consequently, modular plants can be viewed as reproductive mosaics where either ovule number, pollination or resources limit the number of seeds set by different flowers, so that improvements in any of them could increase plant seed production.

## Introduction

**Figure 1:**
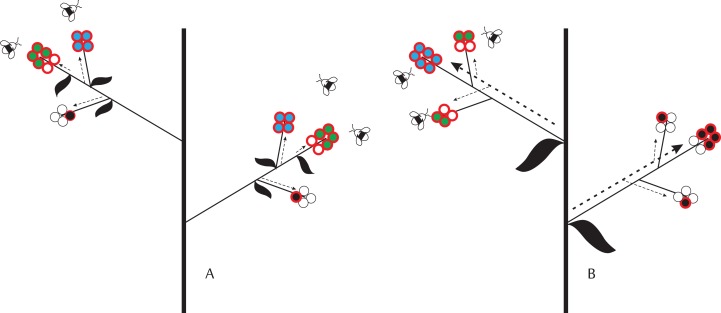
Schematic representation of ovule-, pollination-, and resource-limitation of seed production by individual flowers given different levels of physiological integration, which decreases from (A) (i.e., flower) to (B) (i.e., inflorescence). Circles with a red border represent fertilized ovules. Empty and filled circles identify aborted ovules/embryos and developing seeds, respectively. We assume that final seed size is fixed. Colors identify different limits on seed set: blue—ovule limitation; green—resource limitation; black—pollen limitation. Dashed arrows identify the direction of resource allocation within plants and their thickness represents the intensity of resource allocation: (A) fixed resource allocation to individual flowers/fruits; and (B) fixed allocation per inflorescence, but demand-driven allocation to developing embryos. In (A), all flowers in the inflorescence have enough resources to produce four seeds. Seed set is pollination-limited if flowers receive insufficient pollen to fertilize all ovules and there are sufficient resources for seed development. Seed set is resource-limited when some embryos lack resources for developing seeds (e.g., a flower has six embryos but the resources for producing only four seeds). Ovule limitation of seed set occurs when all ovules are fertilized and sufficient resources are available to develop the embryos into seeds. In (B), flowers with more ovules receive a larger share of seed resources. Given sufficient pollination, the upper limit to seed production in flowers with several ovules is therefore set by ovule number. Flowers with fewer ovules receive a smaller share of resources and, under proper pollination, their seed set is therefore resource-limited. Because of phenology or position, one inflorescence in this plant received fewer pollinator visits. As a consequence, seed set in flowers of this inflorescence is pollination-limited.

Seed production can be affected by a diversity of factors acting during flower and fruit development (Primack, 1987). Some of these can limit ovule fertilization (e.g., pollination quantity and quality), whereas others affect the likelihood of completing seed development (e.g., resource availability, embryo quality, and seed predation; Harder & Routley, 2006). A classical perspective proposes that either pollination or plant resources, for example, carbohydrates, limit seed production (Stephenson, 1981; Knight, Steets & Ashman, 2006). Nevertheless, given the receipt of abundant high-quality pollen and enough resources for developing all fertilized ovules into seeds, the ultimate factor limiting seed set is the number of ovules per flower (Burd et al., 2009; Harder, Hobbhahn & Richards, 2012; Ida, Harder & Kudo, 2015) (Fig. 1). In addition to not considering the contribution of ovule number to seed production, the classical dichotomous perspective on the limits of seed production assumes implicitly or explicitly that resources can move freely within the plant, with all flowers having equal access to resources (Wesselingh, 2007). Challenging this assumption, resource compartmentalization within plant morphological and physiological modules (e.g., inflorescences; Watson & Casper, 1984) has been shown as an important factor determining patterns of reproductive investment into flowers, fruits, and seeds (Guitian, 1994; Ida, Harder & Kudo, 2013, 2015; Dai et al., 2018). Nevertheless, how ovule number, pollination, and resources affect seed production in physiologically-compartmentalized, rather than physiologically-integrated plants, has yet to be explored.

In plants producing flowers with varying number of ovules, ovule number may affect the number of seeds produced by single flowers (Fig. 1). If a relatively constant fraction of the ovules are fertilized and develop into seeds, then seed number will increase with ovule number just because of a numeric, density-independent effect (i.e., considering here as “density” the number of ovules per flower). Alternatively, ovule number can affect the actual fraction of ovules that develop into seeds (i.e., seed set), via density-dependent effects. Given adequate pollination and limiting resources, ovule number can have a negative or a positive density-dependent effect on embryo survival. Under the extreme scenario that a constant amount of resources is allocated to each flower for fruit and seed development (i.e., none physiological integration among flowers, with resources supplied at the individual flower level), a negative density-dependent effect is expected if a higher number of ovules leads to stronger competition for resource access among embryos within a developing fruit (Ganeshaiah & Uma Shaanker, 1994) (Fig. 1A). On the contrary, a positive density-dependent effect is expected if resources or their supply rate are limiting, but flowers are integrated into larger modular physiological units (e.g., inflorescences). In this case, flowers with more ovules and developing embryos within these integrated units could represent stronger sinks for maternal resources than flowers with fewer ovules and embryos (Fig. 1B; Stephenson, 1981; Ida, Harder & Kudo, 2013, 2015). Positive density-dependent effects of ovule number on seed set are expected to originate at the level of plant modularity at which flowers are most physiologically integrated. In a plant where all flowers are physiologically integrated (i.e., absence of physiological modularity), the positive density-dependent effect of ovule number on seed set should occur at the whole-plant rather than, for instance, the inflorescence level. The contrary would be expected if the inflorescence, rather than the whole plant, were the unit at which flowers are most physiologically integrated.

While plant physiological modularity may affect the sign and the level at which the density-dependent effect of ovule number on seed set originates, overall plant resource status could affect the strength of this association. This is so because resourceful plants are able to nourish most developing embryos, thus eroding any relation between ovule number and seed set. Instead, resource-limited plants are able to nourish just a fraction of the developing embryos (Ladio & Aizen, 1999; Ashman & Hitchens, 2000; Medrano, Guitian & Guitián, 2000; Kliber & Eckert, 2004). In this latter case, ovule number per flower can become an important factor determining what fraction of the developing embryos mature into seeds. Therefore, we expect density-dependent effects of ovule number on seed set to increase with increasing resource limitation. Furthermore, even though a density-dependent effect of ovule number on seed set may occur in plant lineages with a variety of gynoecium anatomies, this effect may be particularly apparent in lineages producing flowers with apocarpic ovaries (i.e., multi-pistilate flowers). In these lineages, investment in seed ancillary structures (e.g., fruit flesh) occurs on a single-pistil basis, and, as a result, resource investment into fruit development is expected to increase strongly with the number of ovules when pollination is not limiting (Wetzstein et al., 2013).

Spatial and temporal variation in pollen supply can also affect flower and plant seed set (Wesselingh, 2007; Harder & Prusinkiewicz, 2012), adding a layer of complexity to the relation of seed set to ovule number. As explained above, the classical dichotomous perspective on the factors affecting seed production assumes that resources can move freely within a plant (i.e., complete physiological integration) so that all flowers of an individual are limited by either pollen or resources (Knight, Steets & Ashman, 2006). If this were the case, a resource-driven density-dependent effect of ovule number on seed set should become progressively diluted with increasing pollen limitation as seed set becomes increasingly independent of ovule number. But if a limiting plant resource, for example, carbon, is produced and allocated within relatively autonomous physiological subunits, like flowers or inflorescences, density-dependent effects of ovule number on seed set can persist under a scenario of pollen limitation, as long as some flowers or inflorescences are not limited by pollination (Fig. 1B). Therefore, this modular perspective of plant structure and resource allocation predicts that plants producing many flowers can be seen as mosaics of limiting factors in which seed set in different flowers can be determined alternatively by ovules, pollen or resources, even though one factor can prevail over the others.

**Figure 2:**
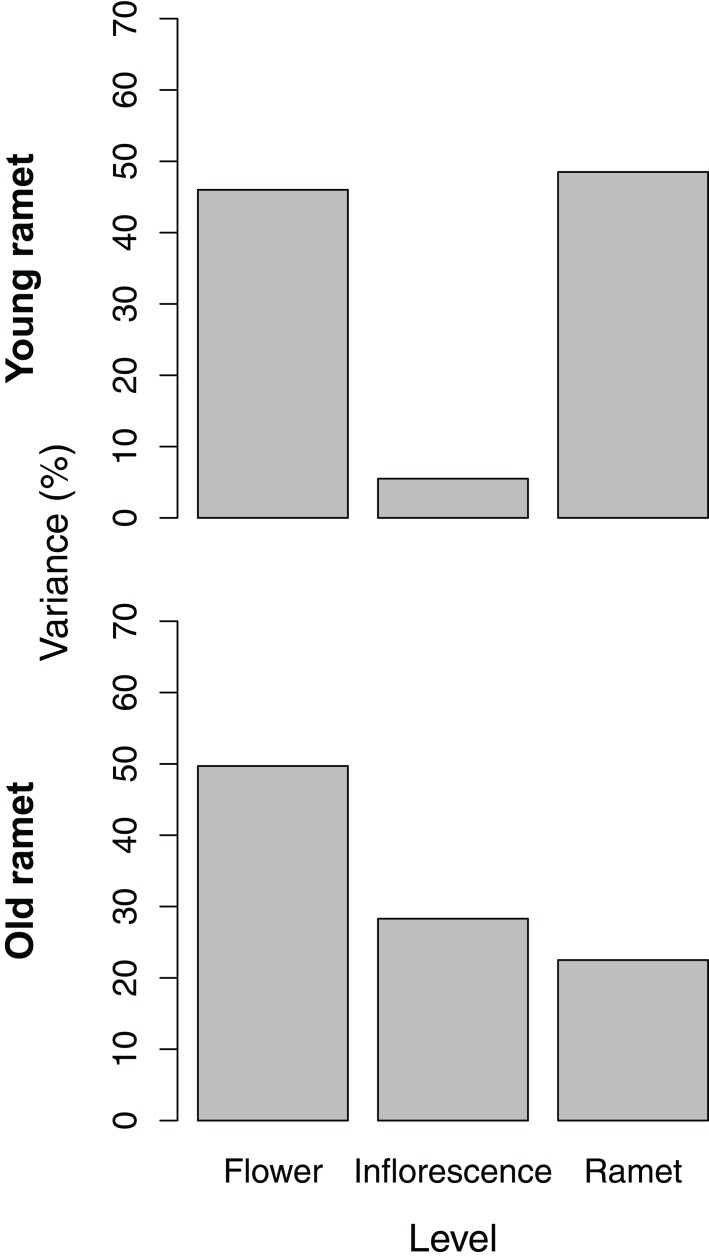
Variance components, as a percentage of the total, of ovule number among flowers within inflorescences, among inflorescences within ramets, and among ramets for young and old ramets.

Here we propose that: (1) ovule number has a density-dependent effect on flower seed set, with the sign of this effect (i.e., negative or positive) and the level at which it originates (i.e., among flowers within inflorescences, among inflorescences within plants, and among plants) depending on the extent of physiological integration among flowers at these different levels (Fig. 1); (2) resource limitation at the whole plant level increases within-plant variation in flower seed set, thereby strengthening the dependence of seed set on ovule number; and (3) a density-dependent effect of ovule number on flower seed set can persist despite some flowers being pollen-limited, as long as the flowers are not physiologically integrated at the whole plant level. We tested these ideas using raspberry as a model system.

Raspberry, *Rubus idaeus* (Rosaceae), is an appropriate species to test hypotheses on the nature of the relation between the number of ovules, resources, pollination and seed set. The first aspect that makes the raspberry suitable for our study is its ovary anatomy. Individual flowers have many uniovulate carpels that, once fertilized, develop into apocarpic fleshy fruits (i.e., polydrupes). Therefore, resource investment into single fruits, and thus fruit size, depends strongly on the number of seeds. Second, this species is highly modular allowing testing hypotheses about how physiological integration among flowers would shape a possible density-dependent effect of ovule number on seed set. As a large proportion of the carbon allocated to a raspberry fruit is fixed by the inflorescence subtending leaf (Waister & Wright, 1989), we expected a high degree of physiological integration among flowers belonging to the same inflorescence. Therefore, any effect of ovule number on flower seed set should be positive and occur at this level. Third, the raspberry’s characteristics, and particularly those of the Autumn bliss variety, which we used in this study, allowed us to address questions about how the relation between ovule number and seed set changes with overall plant resource status. Raspberry plants are clonal with underground rhizomes that produce aerial shoots (hereafter “ramets”). In particular, the studied Autumn bliss variety produces biennial ramets that flower twice, once during their first summer (i.e., young ramets or “primocanes”) and again at the beginning of the following summer (i.e., old ramets or “floricanes”). Old ramets have smaller and more achlorophyllous leaves than young ramets (M. Strelin, 2015–2016, personal observation), and their crop yield is highly constrained by resource competition with the developing young ramets (Waister & Wright, 1989). Finally, although the Autumn bliss variety is fully self-compatible and capable of extensive within-flower self-pollen deposition and autogamous fruit production, insect visitation is needed for the pollination of all pistils in a flower and production of a high-quality fruit (Cane, 2005). This partial-autogamy allowed us testing whether density-dependent effects of ovule number on seed set persist when plants become more pollen limited after excluding pollinators.

In this study, we first explored how variation in ovule and seed number is partitioned among flowers within-inflorescences, among-inflorescences within ramets, and among-ramets. This preparatory step is important to infer patterns of resource allocation to ovule and seed production at different levels of the modular structure of this plant, that is, differential resource allocation would be reflected in high variance components. We then evaluated the occurrence and sign of a density-dependent effect of ovule number on seed set overall and at different hierarchical levels of raspberry modular structure (Objective 1). Since in raspberry most of the carbon allocated for fruit development is fixed by the inflorescence subtending leaf, we predicted high variance components for ovule and seed number, as well as a positive density-dependent effect of ovule number on seed set among flowers within inflorescences. Next, we evaluated whether ovule number, overall resource availability (i.e., young vs. old ramets), and the interaction between these factors affect seed set, and whether this effect becomes diluted or not after excluding plants from pollinators (Objective 2). Since resource shortage increases within-plant variation in seed and fruit investment (see above), we expected that the density-dependent effect of ovule number on seed set was stronger in old than young ramets. Regarding pollination, we did not expect a strong dilution of the density-dependent effect of ovule number on seed set after excluding pollinators (i.e., an interaction between ovule number and pollination treatment) as long as resource allocation among developing fruits did occur within plant modules (i.e., inflorescences). Last, because raspberry produces flowers in cymose inflorescences that have up to three axis orders, we evaluated the possibility that density-dependent effects on seed set could be mediated by architectural effects (i.e., axis or branching order) rather than by ovule number (Objective 3). High abortion of fruits that develop from late-opening flowers or are nourished through thinner vascular connections associated with higher-order inflorescence axes has been observed under diverse scenarios of resource limitation (Ladio & Aizen, 1999; Ashman & Hitchens, 2000; Medrano, Guitian & Guitián, 2000; Kliber & Eckert, 2004). Therefore, this latest assessment is critical to support any argument of a causal link between ovule number and embryo survival.

## Materials and Methods

### Study site

Fieldwork was conducted from December to April of the 2015–2016 austral summer, in a commercial raspberry field in Northwestern Patagonia, Argentina, about 10 km east of the city of San Carlos de Bariloche (41°06′26.3″S; 71°12′11.7″W). The 0.4-ha field was cultivated with the Autumn bliss variety and was managed organically and irrigated artificially. In the NW Patagonia region, the managed honey bees, *Apis mellifera*, and the invasive bumble bee, *Bombus terrestris*, account for >90% of visits to raspberry flowers (Sáez et al., 2014), with the native halictid bee *Ruizanthedella mutabilis* and other native insects accounting for the rest of the visits (Morales, 2009; M. Strelin, 2015–2016, personal observation).

In the southern hemisphere, young ramets flower from January to mid-March, and their fruits mature from February to April. Typically, these ramets are pruned after fruiting and their dormant buds start regrowing the following spring, becoming old ramets. These old ramets flower during December and mature fruits during January.

### Field sampling

We haphazardly chose 25 young ramets produced during the sampling season and 25 old ramets from the previous season distributed over the entire field. Each inflorescence produced by each of these ramets was identified and marked with a paper tag. Each inflorescence was then sketched, mapping the position and branching order of each flower. Flowers were classified based on the order of their subtending floral axes as primary, secondary, or tertiary. Higher order flowers were not included in this study because of reduced sample size. To assess the effect of pollinator visits on seed set, we selected 10 young and 10 old ramets and excluded pollinators by covering them with a wire-framed net mesh fixed to the ground. Pollinator excluded and control ramets were interspersed across the entire field. The other 15 young and 15 old ramets were left open to insect pollination. Fruits were harvested when the polydrupe was bright red and detached easily from the receptacle. Collected fruits were kept under shade for ≤4 h before being transported to the laboratory in San Carlos de Bariloche, where they were frozen at −80 °C until processing. From these fruits, we estimated ovule and seed number per flower based on the number of fully developed and persistent aborted drupelets. The latter can be recognized as thiny hair-like structures among the red, fleshy drupelets. Developed and aborted drupelets of each fruit were counted under a dissecting microscope at 10×. Total ovule number per flower was estimated by adding the number of drupelets and of aborted pistils, and seed set by dividing the number of drupelets by ovule number. A haphazardly-chosen subset of a total of 25 aborted and 25 developed drupelets from fruits of five ramets was dissected to ensure that they contained ovules, embryos or seeds, which was always the case. Raw data used in this study can be consulted in Table S1.

### Data analysis

We first estimated variance components for the number of ovules and the number of seeds (=number of drupelets) among flowers within inflorescences, among inflorescences within ramets, and among ramets. Hierarchical variance analysis followed Gelman & Hill (2006) as implemented in the *lmer* function of R version 2.4.0 (R Core Team, 2017) (library: lme4; Bates et al., 2014). In the case of ovule number, the analysis was carried out for the young- and old-ramet categories, separately. In the case of seed number, this analysis was conducted separately for each of the four combinations of ramet age and pollination treatment.

Generalized linear mixed-effects models (GLMM) were then used to assess the hierarchical level (i.e., among flowers within inflorescences, among inflorescences within ramets, or among ramets) at which ovule number determines the proportion of formed seeds (objective 1); the effects of ovule number per flower, ramet age (young vs. old ramet) and pollination treatment (pollinator exclusion vs. open pollination) on seed set (objective 2); and the direct effect of flower hierarchy within the inflorescence on seed set and its indirect effect via ovule number (objective 3). Analyses considered “ramet” and “inflorescence within ramet” as random effects (Gelman & Hill, 2006). In all cases, we selected the most appropriate random structure based on AIC criterion, following the backwards model selection protocol suggested by Zuur et al. (2009). Models were implemented with the statistical software R version 2.4.0 (R Core Team, 2017) using the *glmmadmb* function (library: glmmADMB; Bolker et al., 2012) (objectives 1, 2 and 3) and the *glmer* function (library: lme4, Bates et al., 2014) (objective 3).

For objective 1, we assessed the effect of each of the three measures of ovule number on seed set per fruit. In addition to each flower’s ovule number, this analysis included the mean number of ovules per flower at the inflorescence and ramet levels. Since the number of ovules of an individual flower includes a component associated with its inflorescence mean, and the inflorescence mean includes a component associated with its ramet mean, ovule number at each level had to be corrected for variation in ovule number included in the level immediately above. Therefore, we first estimated mean values of ovule number per flower at the inflorescence and ramet levels, corrected by the number of observations for each inflorescence and ramet, by means of an intercept-only model, with a random intercept and two nested levels (flowers within inflorescences and inflorescences within ramets). This analysis was performed with the *lmer* function (library: lme4, Bates et al., 2014). The coefficients retrieved for each level are the sample-size corrected means of each inflorescence and ramet. Second, using these values we calculated the difference between the number of ovules of each individual flower and the corrected mean value of ovules per flower of its corresponding inflorescence, and the difference between the corrected mean value of the number of ovules per flower between each inflorescence and its corresponding ramet. In this way, we estimated independent components of ovule number that could be attributed to the individual flower (i.e., the difference in the number of ovules between the flower and inflorescence levels), the inflorescence to which this flower belonged to (i.e., the difference in the number of ovules between the inflorescence and ramet levels), and the ramet to which this inflorescence belonged to (i.e., the mean number of ovules per flower at the ramet level). These three hierarchical, independent variables were included as predictors of seed set using a generalized linear mixed model with a beta-binomial distribution of the dependent variable and a logit-link function. The chosen distribution accounted for both the nature and the presence of over-dispersion in the residuals of the response variable. Our model also considered interactions between plant age and ovule number at each hierarchical level (i.e., flower, inflorescence, and ramet), since plant resource status could presumably affect the relation between ovule number and seed set at any of these levels.

For objective 2, we used backward elimination, as described in Zuur et al. (2009), to assess the relative importance of the studied factors and their mutual dependence on seed set. The initial model included the three studied fixed factors (i.e., ovule number of individual flowers, without accounting for the mean number of ovules at the inflorescence level; ramet age; and pollination treatment) as main predictors and all their interactions. Because of over-dispersion of model residuals and the nature of the response variable (i.e., seed set), the analysis considered a beta-binomial distribution of seed set and a logit-link function. Likelihood-ratio tests of partial effects were conducted with the *Anova* function of the car R library (Fox & Weisberg, 2011).

To address objective 3, we carried out two analyses. The first determined whether ovule number varied with branching order within inflorescences (i.e., primary, secondary, and tertiary). This analysis, which considered a Poisson distribution of the response variable (i.e., ovule number) and a log-link function, included branching order, ramet age, and the branching order × ramet age interaction as fixed effects, and ramet and inflorescence within ramet as random factors. The second analysis, which considered seed set as the response variable, added flower order and its interaction with ramet age to the reduced model from the preceding analysis (i.e., objective 2). Likelihood-ratio tests of partial effects were conducted with the *Anova* function of the car R library (Fox & Weisberg, 2011).

## Results

### Variance partitioning of ovule and seed number

The partitioning of variance in ovule number among flowers within inflorescences, among inflorescences within ramets, and among ramets depended on ramet age. For young ramets, the flower and ramet levels each accounted for ∼45% of the total variance in ovule number, with the remainder associated with inflorescences within ramets (Fig. 2). For old ramets, the flower level accounted for the largest portion of variation (∼50%) in ovule number, whereas the ramet level accounted for the least (∼20%).

**Figure 3:**
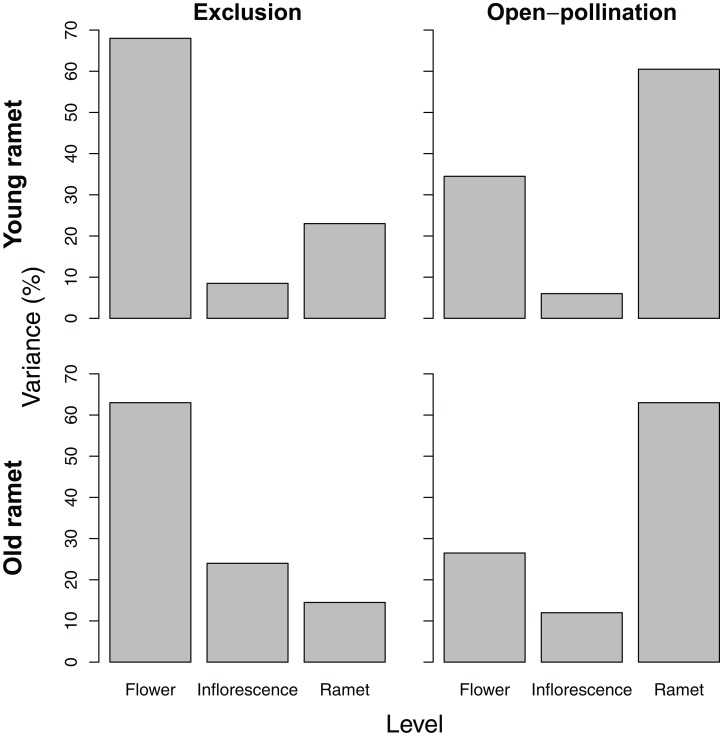
Variance components, as a percentage of the total, of seed number among flowers within inflorescences, among inflorescences within ramets, and among ramets for young and old ramets that were excluded from or exposed to pollinators.

Variance partitioning in seed number per fruit differed depending on ramet age and pollination treatment. For young ramets, variance in seed number paralleled that of ovule number, except that pollinator exclusion greatly reduced the among-ramet component, whereas open pollination increased it. For old ramets, the pattern of variation in seed number reflected that of ovule number only for ramets subjected to pollinator exclusion. For those exposed to pollination, seed number varied the most among ramets (Fig. 3).

### Effect of modularity on the relation between ovule number and seed set (objective 1)

**Figure 4:**
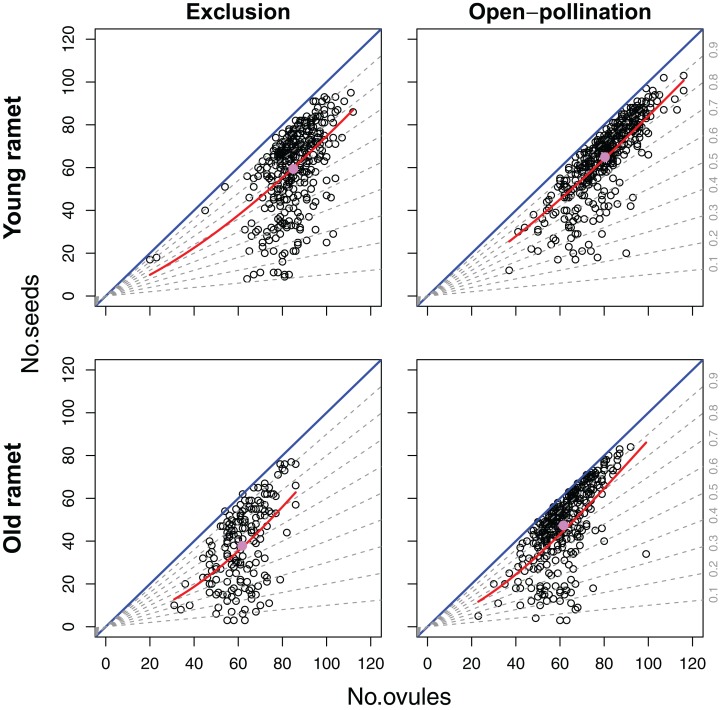
Effects of ovule number, pollination treatment (excluded from or exposed to pollinators) and ramet age on seed set. Blue lines represent complete seed set (intercept = 0, slope = 1). The dashed lines represent specified fixed proportions of ovules that set seed (i.e., density-independent effect of ovule number on seed set). Red curves represent the fitted relation for the best-fit model of variation in seed set. The pink dots represent the average ovule and seed number for each combination of pollination treatment and ramet age.

Even though we found an overall positive effect of ovule number on seed set (Fig. 4), the sign and significance of the relation between ovule number and seed set depended on the level of modularity. Variation in ovule number among flowers within inflorescences had a significant and positive effect on flower seed set (*β* = 0.0201; SE = 0.0054) (*z* = 3.76; *P* < 0.0005). On the contrary, variation in ovule number at the inflorescence level had a negative effect on seed set (*β* = −0.0457; SE = 0.0173) (*z* = −2.63; *P* < 0.01). Seed set was marginally but positively affected by ovule number at the ramet level (*β* = 0.0553; SE = 0.0333) (*z* = 1.66; *P* = 0.0972). There was neither a significant effect of plant age on seed set nor any significant interaction between plant age and the components of ovule number at the different levels of modularity in this model (Table 1).

**Table 1:** Results of partial-effect analyses of the deviance for different models designed to address the three objectives of this study.

Term	Ovule number at different hierarchical levels on seed set (Objective 1)	Reduced model (Objective 2)	Branching order and plant age on ovule number (Objective 3)	Reduced model including branching order (Objective 3)
Intercept	*G*_1_ = 1.80	*G*_1_ = 12.70***	*G*_1_ = 23,726.82***	*G*_1_ = 5.90*
Pollination treatment		*G*_1_ = 9.36**		*G*_1_ = 9.40**
Number of ovules (flower)	*G*_1_ = 14.12***	*G*_1_ = 33.27***		*G*_1_ = 25.26***
Number of ovules (inflorescence)	*G*_1_ = 6.92**			
Number of ovules (ramet)	*G*_1_ = 2.75			
Ramet age	*G*_1_ = 1.29	*G*_1_ = 4.10*	*G*_1_ = 47.51***	*G*_1_ = 2.71
Number of ovules (flower) × Ramet age	*G*_1_ = 1.03	*G*_1_ = 4.20*		
Number of ovules (inflorescence) × Ramet age	*G*_1_ = 2.32			
Number of ovules (ramet) × Ramet age	*G*_1_ = 1.21			
Branching order			*G*_1_ = 40.95***	*G*_1_ = 4.59
Branching order × Ramet age			*G*_1_ = 9.10*	*G*_1_ = 3.05

**Notes:**

The initial model tested the effect of ovule number at different plant levels, and its interactions with ramet age, on flower seed set (Objective 1). A second model involved consideration of the three fixed factors affecting the fraction of formed seeds, i.e., ovule number, ramet age, and pollination treatment, and of all their interactions (Objective 2). A third model tested the effects of branching order, plant age and their interaction on ovule number (Objective 3), whereas a final model added to the reduced model of Objective 2 the effects of branching order and branching order × ramet age interaction (Objective 3).

* *P* < 0.05,

** *P* < 0.01,

*** *P* < 0.001.

### Effects of ovule number, plant age and pollination treatment on seed set (objective 2)

For the 1,282 flowers we examined, an average of 73.3% of a flower’s ovules became seeds, with minimum and maximum values that ranged between 4.7% and 100%, respectively. The best-fitting model explaining variation in seed set included ovule number, pollination treatment, plant age and the ovule number × plant age interaction (Table 1). Of all the deviance explained by the fixed factors, 65.4% was accounted by ovule number, 18.4% by ramet age, 8.0% by pollination treatment, and 8.3% by the ovule number × ramet age interaction. Because of the strong positive effect of ovule number on seed set, the number of seeds, and thus of drupelets per fruit, increased disproportionally with the number of ovules (*β* = 0.02804; SE = 0.00508) (*z* = 5.62; *P* < 0.001). However, in a large number of fruits the total number of ovules per flower imposed a strict upper limit on the number of seeds produced (Fig. 4). Fruits produced by young and old ramets had an average of 62.4 and 44.1 seeds, respectively; with flowers from young ramets showing a seed set, on average, 31.5% larger than flowers from old ramets (*z* = 2.13; *P* < 0.05). Ramet age affected the positive density-dependence of ovule number on seed set, being stronger in old ramets (*z* = 2.17; *P* < 0.05) (Fig. 4). Flowers from plants excluded from and exposed to pollinators produced an average of 52.0 and 56.9 seeds, respectively; with flowers from plants exposed to pollinators showing a seed set, on average, 8.52% larger than that from plants that were excluded (*z* = 2.60; *P* < 0.01). Unlike ramet age, pollination treatment did not significantly affect the shape of the relation between seed set and ovule number (*z* = −1.28; *P* = 0.20) (Fig. 4).

### Effect of branching order and plant age on ovule number and seed set (objective 3)

**Figure 5:**
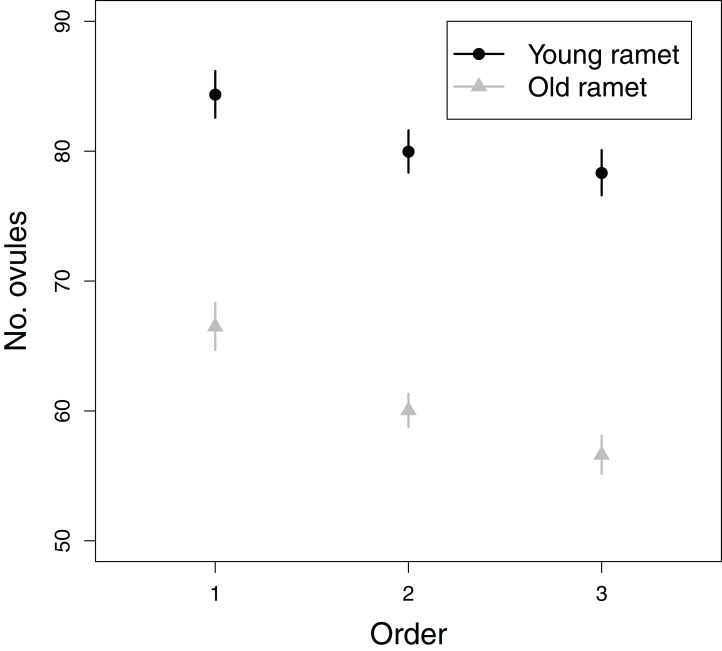
Effects of flower order and plant age on the mean (±SE) number of ovules per flower.

All fixed effects in the first model addressing objective 3, i.e., branching order, plant age and the branching order × plant age interaction, significantly influenced ovule number (Table 1). The number of ovules per flower greatly decreased from first to tertiary-order flowers (Fig. 5). Also, old ramets produced flowers with fewer ovules than young ramets (Fig. 5). The decrease in ovule number with increasing branching order was somewhat weaker in young than in old ramets (Fig. 5). First, second, and third order flowers in young ramets had, on average, 84.4, 80.0, and 78.3 ovules, respectively, whereas in old ramets had 66.5, 60.0, and 56.6 ovules, respectively. In contrast to the effect of flower branching order on ovule number, branching order did not significantly affect seed set after accounting for variation in ovule number (Table 1). Therefore any effect of plant architecture on seed set has to be interpreted as indirect, i.e., via ovule number.

## Discussion

This study reveals the importance of ovule number as an overriding factor affecting the number of raspberry seeds, so that the higher the number of ovules per flower the higher the proportion of ovules becoming seed. Within inflorescences, resources appear to be differentially allocated to developing fruits from flowers with many ovules, which exhibited higher seed set. This allocation pattern was stronger in resource limited plants and persisted despite pollinator exclusion and even after accounting for the branching order of flowers.

### Variation in seed set as a direct consequence of variation in ovule number

Our results indicate a clear distinction in the way resources are allocated to ovules and seeds. Whereas ovule number varied with branching order within the inflorescence (Fig. 5), the proportion of those ovules finally becoming seeds was determined more directly by ovule number rather than branching order (Table 1). Thus, ovule number might reflect the supply rate and total amount of resources provided during flower development; resource supply rate could, in turn, relate to the thickness of the vascular tissue that is expected to vary inversely with branching order (Wetzstein et al., 2013). Instead, seed set seems to be the result of the sink strength of an increasing number of developing embryos associated with a higher number of ovules. Embryos can have high endogenous levels of cytokinin and gibberellin that are involved in their nutrition (Finkelstein, 2010). As a consequence, a larger number of embryos could provide a stronger hormonal signal that would result in a developing fruit obtaining a disproportionate share of available resources (Stephenson, 1981). Therefore, whereas allocation to ovule number seems to be determined by a pure maternal feature like inflorescence architecture, allocation to seeds seems to be under an indirect maternal control through variation in ovule number.

### A modular perspective of the relation between ovule number and seed set

The positive density-dependent effect of ovule number on seed set occurs basically among flowers within inflorescences and suggests that, within well-pollinated inflorescences, flowers with more ovules and embryos become stronger sinks for locally limiting resources (Fig. 1B). Extensive variation in ovule and seed number within inflorescences (Figs. 2 and 3) agrees with the view that differential resource allocation is strongest at this level of the modular organization of a raspberry plant (Guitian, 1994; Wesselingh, 2007; Herrera, 2009). At the inflorescence level, however, there was a negative density-dependent effect of ovule number on seed set, suggesting that inflorescences rely on a relatively constant pool of resources that must be allocated to developing embryos and their ancillary fruit structures. Hence, resources for seed development would become more limiting as the average number of ovules (and ensuing embryos) per flower at the inflorescence level increases. This latter result is consistent with the fact most carbon used for fruit development seems to be fixed by the inflorescence subtending leaf, with little subsidy from photosynthetic structures outside the inflorescence (Waister & Wright, 1989). Therefore, the findings we report here clearly support a modular perspective on how resources are compartmentalized and allocated to seed production based on the strength of source-sink relations associated with variation in ovule number.

### The interplay between ovule number, resources and pollination

Our findings support the hypothesis that plant resource status is an important factor mediating the relation between ovule number and seed set. A reduction in ovule number and seed set observed in old ramets supports our assumption that resources allocated to reproduction are particularly limiting in ramets that have already produced fruit. Furthermore, old ramets exhibited a more marked positive density-dependent response of seed set to ovule number than young ramets (Fig. 4). This finding suggests that the sink effect of developing fruits with many embryos, produced by flowers with many ovules, becomes exacerbated under increasing resource limitation, which results in some fruits with many seeds and others with too few. Even though ovule number here affected seed set independently of branching order (Table 1), the reduced vascularity of higher order flowers (Wetzstein et al., 2013) might also constrain the supply rate of seed resources, thus exacerbating any effect related to ovule number per se. In the raspberry, resource-limited flowers on higher-order branches (which also have reduced ovule number) usually develop into fruit even if a reduced fraction of pistils develops into drupelets. Regulation of seed number in response to changes in the amount of resources through changes in seed set rather than fruit set (Ladio & Aizen, 1999; Ashman & Hitchens, 2000; Medrano, Guitian & Guitián, 2000; Kliber & Eckert, 2004) can be common among species producing apocarpic fruits, like raspberries, because seed resources can be allocated separately to each carpel (Wetzstein et al., 2013). Therefore, increasing resource limitation could increase variability in seed set among fruits (Fig. 4), and exacerbate the positive density-dependent effect of ovule number on seed set.

Resource-driven, density-dependent effects of ovule number on seed set did not diminish with increasing levels of pollen limitation, as expected if whole plants were physiologically integrated. This result challenges the classical and dichotomous view that seed set is either limited by the receipt of pollen or by available plant resources (works cited in Wesselingh, 2007; Knight, Steets & Ashman, 2006). Even under scenarios of adequate natural pollination, variability in pollinator availability in space and time and in flower placement and phenology should result in the inadequate pollination of a fraction, small or large, of all the flowers produced by a plant (Herrera, 2004; Richards, Williams & Harder, 2009). Because of plant structural limitations associated with anatomical and physiological modularity in raspberry (Waister & Wright, 1989), unused resources from poorly pollinated flowers cannot be reallocated to flowers receiving surplus pollen in different branches (Wesselingh, 2007). Therefore, persistence of the resource-driven relation between ovule number and seed set under increasing pollen limitation can then be understood in the context of resource compartmentalization.

### A mosaic of reproductive limits

Considering that all the study factors play a role in limiting seed set in raspberry, we propose that seed production in modular plants can be frequently subject to a mosaic of reproductive limits, with flowers in some modules limited by pollen, others by resources, and others by ovule number. This latter overlooked factor clearly sets a sharp upper limit to flower seed production (Figs. 1 and 4). Even interactions can exist between these factors, as shown here that the magnitude of the density-dependent effect of the number of ovules could depend on the plant resource status. This mosaic of reproductive limits might also replicate at the within-flower level in plants with apocarpic ovaries, a flower sexual morphology that allows differential resource allocation and differential fertilization of ovules in different carpels. According to this integrative perspective, plants should be viewed as mosaics in which different factors, and interactions between them occurring within integrated physiological units, can limit the seed production of different plant modules. This perspective clearly defies the classical dichotomous view of “pollination or resources” as alternative limits of plant seed production (Wesselingh, 2007), and even the one provided by optimality models that predicts simultaneous limitation by both factors (Haig & Westoby, 1988). Instead, our perspective agrees with the view that individual plants can be seen as mosaics exhibiting high intra-individual phenotypic variation because a multiplicity of factors affect independently different modules (Herrera, 2009).

## Conclusion

Ovule number appears to be the overriding, but not the only, factor affecting the number of seeds at the flower level in raspberry. The strong and positive relation between ovule number and seed set in raspberry may relate, to some extent, to the resource allocation patterns of this plant, where resources are supplied mainly at the inflorescence level and investment in pulp or other ancillary structures is strongly dependent on the number of seeds. More generally, this study proposes that several factors (i.e., ovules, pollination, and resources) can determine plant seed set simultaneously in the context of plant physiological modularity. Hence, an improvement in each of these factors will commonly improve reproductive success. Future studies on the limits of plant reproduction should focus on the relative contribution of these different factors (i.e., pollination, resources, and ovules) to limit seed production in individual plants, rather than searching for a single limiting factor. It would be also worth exploring whether the degree of physiological integration among flowers in lineages with different plant architectures and life histories conditions the way ovules, resources and pollination affect seed production. The scenario here proposed, according to which seed production is limited simultaneously by all these factors, might get diluted, resembling the dichotomous response, in lineages with more physiologically integrated plants (e.g., structurally simple and short-lived herbaceous plants). Nevertheless, no one plant can be considered “a bouquet in a vase” (using the metaphor of Wesselingh, 2007). Adopting a modular perspective of plant reproduction, where plants are viewed as mosaics in which different factors and their interactions within integrated physiological units affect seed set, will increase our understanding of the limits on seed production.

## Supplemental Information

10.7717/peerj.5384/supp-1 Supplemental Information 1 Table S1. Raw data used in the analyses corresponding to objectives 1, 2 and 3.

## Additional Information and Declarations

### Data Availability

The following information was supplied regarding data availability: The raw data are provided in Table S1.
